# Systematic review of randomized controlled trials in the treatment of dry eye disease in Sjogren syndrome

**DOI:** 10.1186/s12950-017-0174-3

**Published:** 2017-11-21

**Authors:** Kendrick Co Shih, Christie Nicole Lun, Vishal Jhanji, Bernard Yu-Hor Thong, Louis Tong

**Affiliations:** 1 Department of Ophthalmology, Li Ka Shing Faculty of Medicine, University of Hong Kong, Pok Fu Lam, Hong Kong; 2 Li Ka Shing Faculty of Medicine, University of Hong Kong, Pok Fu Lam, Hong Kong; 3 Department of Ophthalmology, University of Pittsburgh Medical Centre, Pittsburgh, USA; 4 Department of Ophthalmology and Visual Sciences, Faculty of Medicine, The Chinese University of Hong Kong, Shatin, Hong Kong; 5 Department of Rheumatology, Allergy and Immunology, Tan Tock Seng Hospital, Singapore, Singapore; 6 Ocular Surface Research Group, Singapore Eye Research Institute, Singapore, Singapore; 7 Corneal and External Eye Disease Service, Singapore National Eye Centre, 11 Third Hospital Avenue, Singapore, 168751 Singapore; 8 Eye-Academic Clinical Program, Duke-NUS Medical School, Singapore, Singapore; 9 Department of Ophthalmology, Yong Loo Lin School of medicine, National University of Singapore, Singapore, Singapore

**Keywords:** Dry eye, Systematic review, Randomized controlled trials, Sjögren’s syndrome, Keratoconjunctivitis sicca

## Abstract

Primary Sjögren’s syndrome is an autoimmune disease characterized by dry eye and dry mouth. We systematically reviewed all the randomized controlled clinical trials published in the last 15 years that included ocular outcomes. We found 22 trials involving 9 topical, 10 oral, 2 intravenous and 1 subcutaneous modalities of treatment. Fluoromethalone eye drops over 8 weeks were more effective than topical cyclosporine in the treatment of dry eye symptoms and signs; similarly, indomethacin eye drops over 1 month were more efficacious than diclofenac eye drops. Oral pilocarpine 5 mg twice daily over 3 months was superior to use of lubricants or punctal plugs for treating dry eye, but 5% of participants had gastrointestinal adverse effects from pilocarpine, though none discontinued treatment. In contrast, etanercept, a TNF-alpha blocking antibody, administered as subcutaneous injections twice weekly, did not improve dry eye significantly compared to placebo injections. In conclusion, topical corticosteroids have been shown to be effective in dry eye associated with Sjögren’s syndrome. As some topical non-steroidal anti-inflammatory drugs may be more effective than others, these should be further evaluated. Systemic secretagogues like pilocarpine have a role in Sjögren’s syndrome but the adverse effects may limit their clinical use. It is disappointing that systemic cytokine therapy did not produce encouraging ocular outcomes but participants should have assessment of cytokine levels in such trials, as those with higher baseline cytokine levels may respond better.

(229 words)

## Background

Sjögren’s syndrome (SS) is a gradual and progressive autoimmune disease that primarily targets the exocrine glands. It is estimated that the worldwide incidence of Sjögren’s is 7 per 100,000 people, with the highest incidence rates in Europe and Asia, reaching up to 43 per 100,000 people [1]. In view of such statistics, there is a relative lack of evidence-based treatment on Sjögren-related keratoconjunctivitis sicca (KCS).

SS is characterized by the replacement of functional epithelium with lymphocytic infiltrates, leading to gradual gland destruction and impaired exocrine secretions. Impaired glandular excretion results in sicca symptoms such as oral and ocular dryness. Dry eye from SS is more severe than idiopathic dry eye, so treatment modalities that work in idiopathic dry eye may or may not be effective in SS. There are several reviews and meta-analyses concerning the treatment of dry eye in primary SS, such as rituximab and hydroxychloroquine, but evidence for their therapeutic efficacies is weak. [2–4] In order to address these issues, a systematic review is conducted to summarize and analyze the current evidence for the management of SS-related dry eye, focusing on the efficacy and safety of various topical and systemic treatments, published in the last 15 years, in randomized controlled trials (RCT).

## Methods

We searched the Entrez Pubmed database on the 18th of April, 2017 with the key words “Sjögren’s Syndrome”, and the MESH keywords “therapy” and “controlled trial” and found one hundred and 38 entries.

These papers were then manually curated (by KS, CL, LT) to include only those that reported ocular efficacy outcomes (symptoms and signs of dry eye). Among the included articles, only papers published within the past 15 years were eligible, and only papers that recruited patients with primary SS were included. Studies that did not specify the patients to be diagnosed with rheumatoid arthritis, systemic lupus erythematosis or another named autoimmune disease were included as we assumed those participants had primary SS. We only included articles with full text available in English. We allowed control groups in these studies to use either placebo or standard therapy. With these criteria, we narrowed our results to 20 entries in total. The search strategy was further clarified to include alternate spellings of Sjogren’s syndrome, such as “Sjogren”, “Sjogren Syndrome” and “Sjogren’s”. Other MESH keywords such as “therapy” and “management” were also tested, but did not yield any further results. The references of the curated results were further checked for relevant articles.

## Topical or ophthalmic interventions

We found nine reports on topical interventions and all of them were from single-centered studies; two of these assessed steroidal eye drops: fluoromethalone (FML) and clobetasone. These seven studies did not have large sample sizes: the two studies on steroidal eye drops [5, 6] and the study on punctal plugs [7] recruited only 40 patients each, with the other studies having even smaller samples. Only two of these studies [8] [9] were double-masked. All the studies showed some benefit in efficacy outcomes of dry eye symptoms and signs except tacrolimus eye drops, which only improved clinical signs. Topical steroids, autologous serum eye drops, topical non-steroidal anti-inflammatory drugs (NSAIDs), diquafosol sodium eye drops, punctual plugs, and wearing bandage contact lens (BCL) were associated with improvement in both symptoms and signs. All studies were parallel group RCTs, except the study on autologous serum eye drops, which had a crossover design. Table 1 summarizes the key findings from these studies.

**Table 1:** Summary of Randomized controlled clinical trials in the ocular treatment of ocular complications of Sjögren’s syndrome

	Participants^a^	Intervention	Study arms	Duration	Ocular Efficacy Outcomes	Significant findings	Potential bias/ limitation
Lin, T. and L. Gong (2015)	40 patients Group i (20) Group ii (20)	Topical 0.1% FML eye drops 4 times daily	i. 0.1% FML + 0.1% HA ii. 0.5% CsA + 0.1% HA	8 weeks	• OSDI • CFS • Conjunctival goblet cell density • Schirmer I • TBUT	i provided faster improvement than ii evidenced in CFS, OSDI and conjunctival congestion with statistical significance	• Small sample size • Short duration • Open label design
Aragona, P., et al. (2013)	40 patients Group i. (20) Group ii (20)	Topical 0.1% Clobetasone butyrate eye drops 2 times daily	i. 0.1% clobetasone butyrate eyedrop BD + 2% PVP ii. PEG +2% PVP	30 days	• Dry eye symptoms (VAS) • CFS • LGS • TBUT	i showed improvement in symptoms, CFS and conjunctival stain compared to group ii	• Short study period • Small sample size • No mention of drop out rates.
Moscovici, B. K., et al. (2015)	24 patients Group i (14) Group ii (10)	Topical Tacrolimus eye drops 0.03% every 12 h	i. Tacrolimus 0.03% ii. Almond oil placebo	90 days	• CFS • Rose Bengal stain • Schirmer I • TBUT	Rose Bengal stain (*P* = 0.007) and CFS (*P* = 0.008) improved.	• Small sample size • No symptom-based analysis
Noble, B. A., et al. (2004)	16 patients	Topical 50% Autologous serum eye drops 1 bottle daily	Cross-over study Autologous serum 50% (3 months), Lubricant (3 months)	6 months	• Dry eye symptoms (face score) • CFS • Impression cytology • Rose Bengal stain • Schirmer I	Autologous serum is superior to conventional treatment for ocular surface (P < 0.02), and subjective comfort (P < 0.01)	• Small sample size • No participant masking
Aragona, P., et al. (2005)	20 patients Group i (10) Group ii (10)	Topical NSAIDs eye drops 3 drops daily	i. 0.1% indomethacin ii. 0.1% diclofenac	30 days	• Dry eye symptoms (0–3 scoring system) • CFS • Corneal sensitivity • TBUT	Groups i, ii: improved symptoms (day 15) (P = 0.01; *P* = 0.004); mproved corneal sensitivity (day 30) (*P* = 0.04; *P* = 0.005)	• Small sample size • Short study period • Single blinded
Yokoi N., et al. (2016)	17 patients	3% DQS eye drop 1 drop	i. 3% DQS eye drop in one eye ii. AT solution in the fellow eye	15 min	• Dry eye symptoms (VAS) • CFS • Schirmer I • TBUT • TMR	TMR and VAS score improved in DQS treated eyes at 15 min after instillation (*P* < 0.0001) and (*P* < 0.001).	• Short study period • Small sample size
Li, J., et al. (2015)	37 patients Group i (19) Group ii (18)	BCL daily, 3 weeks of continuous wear	i. BCL ii. Autologous serum	6 weeks	• OSDI • CFS • Schirmer I • TBUT	BCL has a lower OSDI than Autologous serum. (P < 0.001) BCL has less CFS than Autologous serum. (P < 0.01)	• Small sample size • Not blinded • Short study period
Qiu, W., et al. (2013)	40 patients Group i (21) Group ii (19)	Punctal Plugs	i. Punctal plugs ii. Artificial tears	3 months	• OSDI • CFS • Schirmer I • TBUT	Schirmer I and TBUT improved in punctal plugs more than Artificial tears (P < 0.001)	• Short study period • Small sample size
Mansour K., et al. (2007)	13 patients	Punctal Plugs	i. Punctal plugs ii. No intervention (on fellow eye)	6–20 weeks	• Dry eye symptoms (0–10 scoring system) • Rose Bengal stain • Schirmer I	Rose Bengal (P < 0.02) and dry eye symptoms (P < 0.01) improved in punctum-occluded eye. No change in Schirmer I.	• Short study period • Small sample size • Not blinded

^a^All participants in these studies from single center except *Hu* et al., which is multi-center study

*BCL* Bandage contact lens

*CFS* Corneal fluorescein staining

*CsA* Cyclosporin A

*FML* Fluoromethalone

*LGS* Lissamine green stain

*NSAIDs* Non-steroidal anti-inflammatory drugs

*OSDI* Ocular surface disease index

*PEG* Polyethylene glycol

*PVP* Polyvinylpyrrolidone

*TBUT* Tear break up time

*VAS* Visual analogue scale

*DQS* Diquafosol sodium

*TMR* Tear meniscus radius curvature

Both the RCTs on the use of steroidal eye drops showed an improvement in both signs and symptoms of dry eye disease, ie. corneal fluorescein staining (CFS) and Ocular Surface Disease Index (OSDI) symptom score. [5, 6] The study on clobetasone lasted only thirty days, and the comparator was artificial tears. In contrast, the comparator for FML eye drops was topical cyclosporine. In studies recruiting SS patients with more severe dry eye, it may not be ethical to use artificial tear as a comparator, since participants would likely need some form of immunosuppressive treatment to meet standard of care. Both studies similarly recruited SS patients with moderate to severe dry eyes.

A newer T-cell immunosuppressant, tacrolimus, reported to be a hundred times more potent than cyclosporine in in-vitro studies, has been evaluated in a RCT. [10] Over a period of 3 months, instillation of tacrolimus 0.03% eye drops twice daily showed improvement in conjunctival and CFS (*P* = 0.008) but not Schirmer I (*P* = 0.014), compared to almond oil eye drops. [8] Unfortunately measurement of dry eye symptoms was not designated as a trial outcome. In the RCT, 11/ 14 (78.6%) of patients randomized to tacrolimus complained of burning sensation. [8] All patients said that burning lasted for 30 minutes after drop instillation, but none stopped the medication. This modality of treatment may be promising if patients can tolerate the associated burning sensation.

Despite the fact that NSAIDs eye drops have been reported to cause corneal melting [11], scientific evidence supports the use of NSAIDs in dry eye. Firstly, NSAIDs reduce pro-inflammatory eicosanoids in the arachidonic acid pathway. Secondly, the NSAID indomethacin has been shown to reduce chemokines and infiltration of lymphocytes into inflamed tissue. There should be caution in the use of some NSAIDs, diclofenac but not indomethacin reduced corneal sensitivity in normal people. [12] Unfortunately, we did not find a RCT that compared NSAIDs with a non-NSAID comparator. One RCT compared instillation of 0.1% indomethacin with 0.1% diclofenac eye drops 3 times daily over 30 days.[13]Both treatments produced an improvement in dry eye symptoms compared to their baseline levels (*P* < 0.01 for indomethacin; *P* < 0.05 for diclofenac). At 30 days, both symptoms and signs (tear breakup time [TBUT] and CFS) were not statistically different between the two types of NSAIDs. At the same time point, corneal sensitivity significantly decreased compared to baseline (*P* < 0.04 for indomethacin; P < 0.04 for diclofenac).

This RCT allowed liberal use of tear substitutes but the actual frequency of use of each type of eye drops was not reported. The observed improvement in dry eye could be partly due to the concomitant use of tear substitutes. In addition, the scoring system for ocular discomfort was not validated. The study did not mention the incidence of stinging or irritation, which had been encountered after the use of topical NSAIDs. Reassuringly, there were no cases of keratolysis. [13]

Autologous serum is a novel form of treatment for severe dry eye. [14] It consists of various plasma proteins that may have a wound healing or immunosuppressive effect in recalcitrant cases of dry eye. [15] The only RCT in autologous serum in SS employed a parallel group crossover design. [16] The investigators concluded that a bottle of autologous serum (50%) daily was superior to conventional treatment for ocular surface dye staining (*P* < 0.02), and subjective comfort (*P* < 0.01) levels (face scale) over a period of 6 months. [16] The major problem with crossover studies in dry eye is that the adequacy of the washout period in between the two treatments is possibly insufficient, as the residual effect of interventions like autologous serum after cessation is unknown. It is unusual to use a face scale in dry eye studies instead of a validated symptom questionnaire. The authors also reported difficulties concerning the logistics of serum extraction and processing, suggesting that implementation in clinical practice will be challenging.

Diquafosol sodium (DQS) is known to increase tear fluid and mucin production on the ocular surface by acting as an agonism of the P2Y2 purinogenic receptor. [17] In this RCT, 17 study subjects were each instilled with one drop of 3% DQS on one randomly chosen eye and compared ocular efficacy outcomes with its baseline. After 15 min, there is a significant improvement in dry eye symptoms (*P* < 0.001) and central lower tear meniscus radius (TMR) curvature measurement (*P* < 0.0001). [18] Although this study shows an increase in aqueous tear volume after instillation of DQS, it cannot reflect any long-term effect DQS potentially has on the treatment of dry eyes.

Lacrimal punctal occlusion is a major form of treatment in dry eye, especially in patients with aqueous tear deficiency. However, there is a potential drawback because retention of tears may be associated with accumulation of pro-inflammatory cytokines in the tear and ocular surface. Both RCTs on the use of punctal plugs were reported to be beneficial compared to conventional treatment. [7, 9] In one RCT, thermosensitive punctal plugs were inserted in the superior and inferior puncta of each eye and showed statistical improvement in terms of OSDI (*P* < 0.001), CFS (P < 0.001) Schirmer I (P < 0.001) and TBUT (P < 0.001). [7] Unfortunately the study did not report the levels of tear cytokines. The other RCT is a uniocular study that inserted silicone punctal plugs in both the inferior and superior puncta on one eye only in each subjects with comparison to their fellow eye. There is statistical significance in Rose Bengal (*P* < 0.02) and dry eye symptoms (*P* < 0.01). [9] However, the sample size is too small and study duration is too short for the assessment of long-term benefits.

Application of BCL is commonly used in clinics to promote wound healing, especially in cases of persistent corneal epithelial defects. [19] The use of contact lens in severe dry eye is usually restricted to scleral or PROSE type hard lenses as opposed to soft BCL. The use of soft diameter BCL is associated with corneal hypoxia and even infective keratitis. Therefore, a RCT is appropriate to determine if BCLs are beneficial in SS. However, it is worth noting that *Li* et al. compared BCL against use of autologous serum eye drops instead of artificial tears. [20] Even then, BCL was found to be superior to autologous serum in terms of dry eye symptoms, ie. OSDI (*P* = 0.014) and clinical sign, ie. CFS (*P* < 0.01). [20] Schirmer I results were not changed between or within the groups after 6 weeks.

The BCL used in the RCT was made of silicon-hydrogel balafilcon A with 36% water content. [20] For obvious reasons, there was no participant masking. It was not clear if there was investigator masking. Interestingly, the participants had not been reported to use concomitant prophylactic antibiotic eye drops in this study. The investigators monitored best corrected visual acuity (BCVA) and showed significant improvement in the BCL group (*P* = 0.003), but not the placebo group. [20] No cases of keratitis were reported. However, the study was only 6 weeks in duration and safety of the intervention arms over a longer period remains uncertain.

## Systemic interventions

### Efficacy

We found 13 articles on systemic interventions for dry eye in SS. All evaluated oral agents except etanercept and rituximab, which were administered by subcutaneous and intravenous injection respectively. Two [21, 22] out of 13 studies have large sample sizes (above a hundred and fifty patients), and four of them are multi-centered RCTs. [21, 23] Studies with oral pilocarpine and doxycycline recruited only female participants. [24, 25] All studies were double-masked except for two. [25, 26] In summary, oral pilocarpine, cevimeline, lactoferrin, a traditional Chinese medicine (TCM) herb and linoleic acid/ gamma linoleic acid (5/13 systemic modalities) were found to be more effective than placebo or artificial tear in the treatment of dry eye. [22, 23, 25–28] Table 2 summarizes the key findings from these studies.

**Table 2:** Summary of Randomized controlled clinical trials in the systemic oral treatment of ocular complications of Sjögren’s syndrome

Ono, M., et al. (2004)	60 patients Group i (20) Group ii (21) Group iii (19)	Oral Cevimeline 20 mg or 30 mg 3 times daily	i. Placebo ii. Cevimeline	4 weeks	• Dry eye symptoms (VAS) • CFS • Rose Bengal test • Schirmer I • TBUT	Cevimeline 20 mg: Improvement in symptoms, Rose Bengal test, CFS, TBUT compared with placebo.	• No drop outs mentioned
Petrone, D., et al. (2002)	197 patients Group i (70) Group ii (65) Group iii (62)	Oral Cevimeline 15 mg or 30 mg 3 times daily	i. Placebo ii. Cevimeline	12 weeks	• Dry eye symptoms (VAS) • Schirmer I	Cevimeline 30 mg: Improved in VAS (*P* = 0.045) and Schirmer (*p* = 0.043)	• Short study period
Yoon, C. H., et al. (2016)	26 patients Group i (11) Group ii (15)	Oral HCQ 300 mg daily	i. HCQ, ii. Placebo	12 weeks	• OSDI • CFS • Schirmer I • TBUT	All outcomes are not significant between i and ii at 12 weeks.	• Small sample size • Short study period • Insufficient washout period
Gottenberg, J. E., et al. (2014)	120 patients Group i (56) Group ii (64)	Oral HCQ 400 mg daily	i. HCQ ii. Placebo	48 weeks	• Dry eye symptoms (VAS)	No improvement in VAS between i and ii	• Short study period • Ocular outcome is too subjective • No drop outs mentioned
Sankar, V., et al. (2004)	28 patients Group i (14) Group ii (14)	Etanercept 25 mg twice weekly subcutaneous	i. Etanercept ii. Placebo	12 weeks	• Dry eye symptoms (VAS) • LGS • Schirmer I	No difference in ocular outcomes between i and ii	• Short study period • Small sample size
Tsifetaki, N., et al. (2003)	85 patients Group i (29) Group ii (28) Group iii (28)	Oral pilocarpine 5 mg 2 times daily	i. Pilocarpine ii. Artificial tears iii. Inferior lacrimal puncta occlusion	12 weeks	• Dry eye symptoms (VAS) • Rose Bengal stain • Schirmer I	i improved in symptoms and Rose Bengal stain (P < 0.05) compared with ii and iii	• Short study period • All female subjects
Dogru, m., et al. (2007)	10 patients Group i (3) Group ii (7)	Oral Lactoferrin 270 mg daily	i. Oral lactoferrin ii. Placebo	1 month	• Dry eye symptoms (VAS) • Corneal sensitivity • Schirmer I • TBUT • Tear lipid thickness • Vital staining scores	Significant improvement with Corneal sensitivity, tear lipid thickness and TBUT after 1 month of treatment	• No masking of investigator • Small sample size • Short study period
Seitsalo, H., et al. (2007)	22 patients	Oral doxycycline 20 mg 2 times daily	i. Low dose doxycycline ii. Placebo	10 weeks	• Dry eye symptoms (VAS)	No difference between i and ii in terms of VAS	• Small sample size • No objective signs reported • Cross-over study • All female subjects
Pillemer, S. R., et al. (2004)	23 patients Group i (10) Group ii (13)	Oral DHEA 200 mg daily	i. DHEA ii. Placebo	24 weeks	• Dry eye symptoms (VAS) • Schirmer I • LGS	No differences between i and ii for dry eye symptoms or Schirmer I.	• Small sample size • Limited ocular outcome measurements
Hu, W., et al. (2014)	240 patients Group i (160) Group ii (80)	Oral ShengJinRunZaoYangXue granules 200 g daily	i. ShengJinRunZaoYangXue granules ii. Placebo	6 weeks	• Dry eye symptoms (11 point-Numerical Rating Scale) • Schirmer I	Schirmer I improved in i (*P* = 0.03 for left eye, *P* = 0.02 for right eye)	• Only 1 objective ocular outcome • Short study period
Aragona, P., et al. (2005)	40 patients Group i (20) Group ii (20)	Oral Linoleic acid (omega-6 essential FA) 112 mg + γ-linolenic acid 15 mg daily	i. Omega 6 essential fatty acid ii. Placebo	1 month	• Dry eye symptoms (0–3 scoring system) • CFS • Schirmer I • TBUT • PGE1 (ELISA)	Significantly less symptoms in i compared to ii, reduced CFS in i than ii	• Small sample size • Short study period
Brown, S., et al. (2014)	110 patients	Intravenous Rituximab 1000 mg	i. Rituximab ii. Placebo	48 weeks	• Dry eye symptoms (VAS) • Schirmer I	No significance between i and ii for dry eye symptoms and Schirmer I	• Small sample size
Devauchelle-Pensec, V., et al. (2012)	122 patients	Intravenous Rituximab 1000 mg	i. Rituximab ii. Placebo	24 weeks	• Dry eye symptoms (VAS) • Schirmer I	No significance between i and ii for dry eye symptoms and Schirmer I	• Small sample size

*CFS*, Corneal fluorescein staining

*DHEA* Dehydroepiandrosterone

*ELIZA* Enzyme linked immunesorbent assay

*HCQ* Hydroxychloroquine

*LGS* Lissamine green stain

*OSDI* Ocular surface disease index

*PGE1* Prostaglandin E1

*TBUT* Tear breakup test

*VAS* Visual analogue scale

Pilocarpine is a muscarinic agonist that stimulates secretion of saliva, aqueous tear from lacrimal glands, conjunctival epithelium and mucin from goblet cells. [29] Administration of 5 mg of pilocarpine twice daily, for 12 weeks improved dry eye symptoms and Rose Bengal dye staining compared to the combination of artificial tears and inferior puncta occlusion. [25] The investigators did not clarify whether other medications such as systemic immunosuppressant were used or if a washout period was implemented.

Cevimeline is a parasympathomimetic drug that acts like pilocarpine, except that it has a longer serum half-life. [30] Two RCTs [22, 28] showed that treatment with cevimeline compared to placebo improved symptoms (Visual analog scale) and signs (Schirmer I) of dry eye. Oral cevimeline (15 mg) thrice daily is superior to placebo in terms of symptoms and signs of dry eye. There is some controversy as to whether a higher dosage of 30 mg thrice daily would provide further benefit. Although *Petrone* et al. is a randomized study, participants who received 15 mg had more severe dry eyes than those with 30 mg. Due to the differential severity at baseline, the interpretation of results is more difficult. In these studies the rationale for the dosages used was not explained, and especially, why doses lower than 15 mg were not considered.

Elevated levels of plasma matrix metalloproteinase (MMP) have been reported in primary SS patients, [31] suggesting that these molecules could be therapeutic targets. Doxycycline is known for its bacteriostatic properties, but at low doses, it suppresses MMPs. [32] Oral doxycycline 20 mg twice daily for 10 weeks was not found to be better than placebo in terms of relieving dry eye symptoms. [24] Unfortunately the signs of dry eye were not evaluated.

Tumor necrosis factor alpha (TNF-α) is an immune mediator that has been targeted in the treatment of inflammatory diseases. However, one such therapy, subcutaneous etanercept twice weekly for 3 months, did not improve symptoms and signs (Schirmer I, lissamine green staining) of dry eye compared to placebo. [33] It is possible that the drug was unable to reach target tissues or a larger dose may be needed.

Dehydroepiandrosterone (DHEA) is a steroid hormone that is currently under study for its role in autoimmune diseases. Patients with primary SS have been found to have dysfunctional hypothalamic-pituitary-adrenal axes. [34, 35] DHEA could be converted into testosterone or estrogen intra-cellularly and replenish the suppressed hormonal levels in primary SS patients, thus improving sicca symptoms. [34, 35] However, daily administration of 200 mg DHEA for 6 months did not improve dry eye symptoms and signs (Schirmer I and lissamine green staining) compared to placebo. [36] Unfortunately plasma DHEA levels were not measured on study subjects, and patients with lower DHEA may potentially benefit more.

Lactoferrin is an interesting modality of treatment. First, lactoferrin is a major tear protein whose concentration is found decreased in dry eye. [37] Second, lactoferrin could modulate oxidative stress and regulate microbes. [38] *Dogru* et al. conducted a 1-month 2-group crossover RCT evaluating oral lactoferrin capsules (270 mg/day) with a small sample size. [26] Lactoferrin improved corneal sensitivity (*P* < 0.01), TBUT (P < 0.01) and tear film lipid layer thickness (P < 0.01) when compared to placebo. [26] Unfortunately, the beneficial effect was not sustainable a month after cessation of lactoferrin. The type of lactoferrin (recombinant or bovine) and bioavailability after oral intake were not reported, and tear lactoferrin levels were not determined. [26] Clinicians should be cautious about applying such results to clinical practice due to a lack of mechanistic investigations in relation to the results from this study. It remains to be seen whether oral administration of lactoferrin has an indirect beneficial effect on the ocular surface through modulation of gut microflora, or whether it exerts effects directly on the tear film.

Hydroxychloroquine (HCQ) is a disease modifying anti-rheumatic drug. It can antagonize toll-like receptors 9 and 7 to inhibit the production of inflammatory markers such as IL-6, known to be overexpressed in SS. [39] In a 12-week RCT on primary SS patients that compared the effect of 300 mg/day oral HCQ and placebo. [40] HCQ did not improve symptoms (OSDI) and signs (TBUT, Schirmer I and CFS) of dry eye, compared to placebo. [40] A longer study (24-week) with a higher dose of HCQ (400 mg/day) still did not improve symptoms (Sicca Symptoms Inventory) and signs (TBUT and Schirmer I). [21] However, in these studies, it was unclear if other immunosuppressants were stopped prior to the use of HCQ.

Rituximab is a monoclonal antibody that targets human CD20 on B-cells. Recently, it has been extensively researched for its potential therapeutic use on treating symptoms and signs of Sjogren’s syndrome as a target therapy. [41] Two large multi-centered, double blind, RCTs (TEARS, TRACTISS) evaluated the efficacy of administrating 1 g of Rituximab intravenously against placebo on patients with primary SS. Ocular symptoms and signs (only Schirmer I) were measured in both studies as secondary outcomes. There was no statistical significance on the endpoints of ocular dryness and Schirmer I in both studies. [42–44] They both concluded insufficient evidence to support the use of Rituximab in most patients with Sjogren’s syndrome. TEARS recruited subjects who were newly diagnosed with primary SS, so low disease activity at baseline was a potential a limiting factor for statistically insignificant results. [44]

Various modalities of TCM have been evaluated in dry eye based on the restoration of ‘yin deficiency’ in specific organs. [23] SS-related sicca symptoms and Schirmer I were evaluated after ingestion of a mixed Chinese herb: ShengJunRunZaoYangXue granules 200 g/day for 6 weeks. [23] These outcomes were improved compared to placebo. Apart from Schirmer I, no other clinical tests of dry eye had been performed. In the study, patients were permitted to continue with other systemic medications such as oral prednisolone, HCQ and other immunosuppressive drugs provided they had been commenced at least 3 months prior to randomization. Nevertheless, the number of such patients included in the study was not reported. Limited knowledge on the molecular mechanisms and targets limits wider adoption of such treatments in routine clinics.

Gamma-linolenic acid (GLA) is an essential omega-6 fatty acid shown to have anti-inflammatory effects in chronic inflammatory diseases. [45] GLA is metabolized into dihomo-γ-linolenic acid (DGLA), an immediate precursor of prostaglandin-E (PGE), an eicosanoid known for its inflammation modulating abilities. [46] A study has found an inverse association between GLA levels and levels of anti-SSA/anti-Ro antibodies. [47] *Aragona* et al. conducted a double-masked RCT using oral consumption of omega-6 essential fatty acids (127 mg) compared to placebo for 1 month. [27] Symptoms of dry eye and CFS were improved at the end of treatment and 15 days after stopping treatment. Interestingly, tear PGE levels (*P* < 0.01) increased compared to baseline, but showed a significant decreasing trend by 15 days after cessation of treatment. [27]

### Safety

In any systemic treatment, safety is of major concern, especially if the treatment is chronic. Though useful, pilocarpine has been found to be associated with the following adverse effects: 4/85 (4.7%) of patients reported mild headache, nausea, vomiting and sweating, though none discontinued treatment. [25] 27/60 (45%) of patients experienced gastrointestinal symptoms from oral cevimeline. [28] In another study, 162/197 (82.2%) of such patients experienced headache, increased sweating, abdominal pain or nausea, though the intensity of these effects was mild. [22]

In patients treated with HCQ, the prevalence of serious adverse effects was not higher than the placebo group, with 5/56 (8.9%) and 7/64 (10.9%) respectively, and these effects were likely unrelated to the use of HCQ. [21] When participants received 24 subcutaneous etanercept injections over 12 weeks, no cases of infection were reported, but 2 patients experienced injection-site reactions. [33] In the TCM study, herbal treatment was associated with adverse effects in 23/160 (15.6%) patients, compared to 8/80 (10.0%) in the placebo group. [23] Two cases of serious adverse events were reported in herbal group, but they were likely to be due to SS- associated autoimmune liver disease rather than the herbal treatment. [23]

## Conclusions

This systematic review analyzed 22 RCTs on the various treatment options for Sjögren-related keratoconjunctivis sicca. Although most of these studies are relatively small, and the papers did not comply with all the Consolidated Standards of Reporting Trials (CONSORT) requirements for reporting RCTs [48], there were encouraging results for a few topical and systemic modalities. Topical corticosteroids, oral pilocarpine and cevimeline have been shown to improve symptoms and signs of dry eye. Interestingly, the use of TCM herbs improved Schirmer I results in a multi-centered, double-masked RCT.

The classification criteria for primary SS has been evolving in the past decade; the latest being the American College of Rheumatology (ACR) and European League Against Rheumatism (EULAR) 2016 classification [49], which brings together the earlier ACR 2012 [50] and American European Consensus Group (AECG) 2002 [51] classification. This has been validated and aims to facilitate classification of patients with early SS and those with predominantly systemic disease [52]. While upcoming studies will benefit from adopting 2016 classification criteria, it is important to note that a number of ongoing trials still use the 2002 AECG criteria and thus remain important in SS research. In studies on dry eyes, baseline plasma DHEA level should be defined clearly prior to DHEA supplementation, and lactoferrin levels should be measured prior to lactoferrin treatment. Furthermore, the severity of dry eye in recruited subjects should be more clearly reported, since SS patients demonstrate a big range of dry eye severity [53].

Longer study periods are necessary to monitor toxicity for systemic modalities. The studies in this review were very short. Ideally, most studies on systemic drugs should focus on long-term outcomes [21], unless the systemic toxicity has been assessed in previous studies.

In conclusion, this systematic review showed several promising treatments that have clinical implications for treatment of SS-related dry eye. Some treatment options, eg. topical NSAIDs, should be further studied with double-masked RCTs, larger sample sizes and stricter selection criteria.

## Abbreviations

BCL: Bandage contact lens
BCVA: Best corrected visual acuity
CFS: Corneal fluorescein staining
FML: Fluoromethalone
NSAIDs: Non-steroidal anti-inflammatory drugs
OSDI: Ocular Surface Disease Index
RCT: Randomized controlled trials
SQS: Diquafosol
SSS: Jögren’s syndrome
TBUT: Tear breakup time
TMR: Tear meniscus radius
TCM: Traditional Chinese medicine
MMP: Matrix metalloproteinase
TNF: Tumor necrosis factor alpha
DHEA: Dehydroepiandrosterone
HCQ: Hydroxychloroquine
GLA: Gamma-linolenic acid
DGLA: Dihomo-γ-linolenic acid
PGE: Prostaglandin-E
CONSORT: Consolidated Standards of Reporting Trials
ACR: American College of Rheumatology
EULAR: European League Against Rheumatism
AECG: American European Consensus Group
